# Investigation of grief and posttraumatic growth related to patient loss in pediatric intensive care nurses: a cross-sectional study

**DOI:** 10.1186/s12904-023-01316-z

**Published:** 2023-12-07

**Authors:** Meral Turgut, Hatice Yıldız

**Affiliations:** 1 Pediatric Intensive Care Unit, Aydın Nazilli Public Hospital, Aydın Nazilli Public Hospital, Yeşil District, 622 Street, No: 2 Nazilli, Aydın, 09100 Turkey; 2 Nursing Faculty, Pediatric Nursing Department, Aydın Adnan Menderes University, Central Campus University Variant Cad. No. 98 Efeler, Aydın, Turkey

**Keywords:** Disenfranchised grief, Grief, Nurse, Pediatric Intensive Care Unit, Posttraumatic growth

## Abstract

**Background and aim:**

Pediatric Intensive Care Units (PICUs) are clinical settings where patient loss is frequently experienced. A sense of professional grief and posttraumatic growth in nurses who have experienced patient loss has a significant impact on psychological and physical health, work satisfaction, turnover rates, as well as on personal and professional relations, and employee loyalty. The aim of this study was to investigate grief and posttraumatic growth in PICU nurses and to examine related factors.

**Methods:**

The study is of cross-sectional design and was conducted with 200 nurses who were working in 87 PICU’s around Turkey during the period March 30 - June 30, 2021. Data were collected with a Descriptive Information Form, the Texas Revised Inventory of Grief (TRIG), the Posttraumatic Growth Inventory (PTGI), and an open-ended question asking for suggestions as to how nurses can cope with loss. Online questionnaires were used in the data collection. Descriptive statistics, the student t-test, one-way analysis of variance, and post-hoc tests were employed in the analysis of the data.

**Results:**

The nurses’ mean scores were 49.425 ± 10.868 on TRIG and 61.450 ± 24.934 on PTGI. A negative weak correlation was found between the intensity of the nurses’ grief and their posttraumatic growth (r = 0.144, p = 0.041). Receiving training on dealing with a patient’s terminal stage (t=-2.688, p = 0.001), feeling comfortable about providing the patient’s care (t = 2.624, p = 0.009) and providing the family with emotional support during patient care (t = 1.979, p = 0.049), and the presence of supporting health professionals reduced levels of grief (t = 2.797, p = 0.000). Being a woman (t = 3.299, p = 0.001), willingness to work in the unit (t=-3.219, p = 0.002), and being given enough time to accept the loss (t = 3.986, p = 0.000) were correlated with higher levels of posttraumatic growth. The nurses most commonly wanted more time allotted to recuperate after a loss (n = 35) and professional support (n = 22).

**Conclusions:**

Nurses experience a moderate sense of grief after a patient’s loss. As levels of grief decrease, posttraumatic growth increases. Healthcare administrators and future researchers can benefit from these findings when planning supportive interventions to help nurses cope with their feelings of grief and achieve posttraumatic growth.

**Strengths and limitations:**

A limitation of the study is that it was conducted only with nurses who were Association members.

## Background

Although many diseases can now be treated thanks to the advances in technology and medical applications, children under the age of 5 in Turkey still lose their lives at a rate of 11.2 per mille; most of such losses occur in hospital intensive care units [1]. Because of this, health workers and nurses who spend significant time with patients are among those who suffer the loss [2]. Pediatric nurses take on a unique caregiving role with vulnerable patients, forming special bonds with both patients and their families in performing their jobs [3]. They especially bond with children and their families during the period of terminal care [4]. This relationship may expose them to intense and repetitively negative experiences related to grief when one of their patients is terminally ill or dies. [5]. Pediatric nurses play an active role in sufficiently informing a family about their child’s last moments, carrying out end-of-life duties for the child, delivering the deceased child to the family, and providing emotional support to the grieving parents after the loss [6]. Such situations inevitably cause nurses to be frequently affected by a child’s death, leading them into a deep sense of loss and feelings of grief [6–8]. The literature relates that grief is a common emotion that nurses experience, one that negatively affects their psychological and physical wellness, satisfaction with work, turnover rates, personal and professional relationships as well as employee loyalty to a great degree [6, 7, 9–14].

At the same time, challenging life experiences such as this that can result in adverse psychological outcomes can also lead to positive developments such as posttraumatic growth [15]. The word “trauma” refers to the significant and powerful distress affecting the psychological and physical health of individuals [16]. The Diagnostic and Statistical Manual of Mental Disorders (DSM-5; American Psychiatric Association [APA], 2013) defines traumatic experiences as those related to injury, exposure to sexual abuse, a real death or the threat of death or having a close encounter with death, witnessing such incidents, or learning that a close friend or relative has experienced such an event [17]. At the same time, DSM-5 states that even hearing that another person has experienced a severe trauma is enough to induce symptoms of traumatic stress [18].

Pediatric nurses are more susceptible to trauma after a patient’s loss than nurses working in other clinics because their patient is a child [19]. It was reported in a study [20] conducted with pediatric intensive care unit nurses that 68.8% of nurses experience posttraumatic growth. Many of the participants said that they had experienced posttraumatic growth after the loss of a child or after conflicts they were having with their colleagues. The results of the study show that occupational traumatic experiences at work, can positively affect post-traumatic development.

While the grief process has negative effects such as depression, anxiety, fatigue and burnout, which are generally associated with grief, it also has potential benefits such as post-traumatic growth [21]. This is why it is clear that singularly focusing on nurses’ levels of grief is insufficient in terms of understanding the effects of patient loss. No research in the literature was encountered that compositely examined the period of grief and posttraumatic growth of pediatric nurses following the loss of a patient.

It is important to know that knowledge on the grief, posttraumatic growth and related factors influencing pediatric intensive care nurses who frequently face the death of a child would be of help in identifying their needs for support. Becoming aware of these needs may play a significant role in reducing nurses’ withdrawal from critical units, increasing their satisfaction and welfare, and finally may make a difference in the way of improving the quality of care provided to children and their families.

This study was conducted to examine the grief process in Pediatric Intensive Care Unit nurses after the loss of a patient as well as the experience of posttraumatic growth and related factors.

## Methods

### Design and setting

This analytical cross-sectional study was conducted in 87 different PICU’s around Turkey.

### Sample

The study universe consisted of 500 Turkish nurses who were members of the Pediatric Emergency Medicine and Intensive Care Association. The Association is a national association of Turkish health professionals whose members are actively working in the field of pediatric emergency medicine or pediatric intensive care. G-power analysis was used for sample calculation and the design effect was accepted as 0.10, the study was completed with 200 nurses. Study inclusion criteria were working as a nurse at PICU and volunteering to participate. The exclusion criterion was not having experienced the loss of a patient in the last six months.

### Instruments

Data were collected with the Descriptive Data Questionnaire, the Texas Revised Inventory of Grief, and the Posttraumatic Growth Inventory.

#### Descriptive data Questionnaire

Besides containing data on the sociodemographics and professional experience of the pediatric intensive care nurses, this form consisted of a total of 16 questions, 15 of which related to the participants’ experiences with loss, including the number of patients who died in the unit, data as to the terminal and grieving periods, and the level of comfort the participants experienced while caring for the dying patient and his/her family. The 16th item was an open-ended question on what kind of approach and practices the participant thought would be helpful during their period of loss. The questionnaire was developed by the researchers in light of the literature [6, 8–22].

#### Texas revised inventory of grief (TRIG)

This inventory was developed by Faschingbauer et al. [23] to measure and assess the intensity of the grief reaction following the death of a loved one. The instrument was adapted to Turkish by Yıldız and Cimete [24]. It is made up of 21 items that are divided into two subscales pertaining to past behavior (8 items) and present emotions (13 items). The score obtained on the past behavior scale is used to assess the intensity of early grief, while the score on the present behavior scale helps to evaluate long-term grief. Since the aim of this study was to assess present grief, the 13-item scale of present emotions was employed. The range of possible scores on this scale varies between 13 and 65; higher scores indicate diminished intensity of grief. In studies similar to the present one using comparable assessment methods, mean scores on the Present Emotions subscale corresponded to having experienced severe grief up until a score of 39 (above the 50th percentile); scores below the 50th percentile were assessed as experiencing a moderate or low level of grief [25, 26]. Cronbach’s alpha value for the Texas Revised Inventory of Grief in this study was 0.89.

#### Posttraumatic growth inventory (PTGI)

Tedeschi and Calhoun [15] developed this instrument to measure the positive growth that individuals achieve after a traumatic experience. The instrument is a 21-item 6-point Likert type of scale. Possible scores on the scale vary between 0 and 105; higher scores indicate greater posttraumatic growth. The Turkish adaptation of the PTGI was produced by Dürü [27]. Cronbach’s alpha value for the scale in this study was found to be 0.96.

### Data collection

Data were collected in five months (March 30, 2021 - August 30, 2021) after permission for the study was obtained from the Intensive Care Association administration and the ethics committee. The data collection instruments were converted into online questionnaires via Google Forms. The forms were sent to pediatric intensive care nurses working in 87 different institutions throughout Turkey via email or WhatsApp channels. Before filling out the forms, the participants were informed about the purpose of the study, and their online consent was received. Participants who did not respond to the questionnaires received two reminders, one week apart, and were reinvited to join in the study. The participant link was kept accessible until the sample size was reached. Participants were not allowed to complete more than one survey questionnaire.

### Data analysis

Statistical data were analyzed with the SPSS 22.0 program. Frequencies and percentage analyses, and mean and standard deviation analyses were used to assess the nurses’ descriptive characteristics, their experiences with loss, and their scale scores. Kurtosis and Skewness measurements were used to determine whether the data were normally distributed. Since the kurtosis and skewness values of the variables were between + 1.5 and − 1.5, the data were accepted as in normal distribution [28]. The differences between the nurses’ scale scores according to their descriptive characteristics and experience with loss were evaluated with the student’s t-test, the one-way analysis of variance (ANOVA), and the post hoc test (Tukey, LSD). Statistical significance was accepted as p < 0.05.

The correlations between the nurses’ scale scores were examined with correlation and regression analyses. Assessments of correlation coefficients (r) were made as follows: 0.00-0.25 very weak; 0.26–0.49 weak; 0.50–0.69 moderate; 0.70–0.89 strong; and 0.90-1.00 very strong [29].

In light of other similar studies, G-Power Analysis was used to calculate sample size, the resulting calculations amounting to 179 with effect size = 0.25 (moderate), power (1-beta) = 0.95 and alpha = 0.05 [30].

### Ethical and research approval

Ethics Committee approval was received from the Aydın Adnan Menderes University Health Sciences Institute Noninterventional Clinical Research Ethics Committee (Approval No. 2021/010; Approval Date: 26.02.2021). Permission for sending out the questionnaires to the nurses was obtained from the Pediatric Emergency Medicine and Intensive Care Association. Permission for the use of the instruments was collected from the developers of the validity and reliability studies of the Turkish versions. The participants provided their informed consent. Only the researchers had access to the data.

## Results

### Participants’ sociodemographic characteristics

The sample consisted of 82% female nurses with an average age 32.84 ± 7.69 years, 54% were married, and 49% had children (as shown in Table 1). Of the participants, 72.5% had bachelor’s degrees and 55.5% had been working in the PICU for more than 3 years. Among the nurses, 77.5% were working in the units willingly.

**Table 1:** Nurses’ sociodemographic characteristics and their experience with loss (*N = 200*)

Variables	n (%)	Variables	n (%)
Age (Mean: 32.840 ± 7.692)		Loss of their own child (n = 98)*	
21–30	96(48)	Yes	33(33.7)
31–40	61(30.5)	No	65(66.3%)
Over 40	43(21.5)	Number of children lost in last 6 months	
Gender		1–10 children	138 (69.0)
Female	164(82.0)	11–20 children	36 (18.0)
Male	36(18.0)	21 or more children	26 (13.0)
Civil status		Education on terminal period and grief	
Married	108(54.0)	Yes	44(22.0)
Single	92(46.0)	No	156(78.0)
Has children		Feeling comfortable with looking after a terminal child	
Yes	98(49.0)	Comfortable	40(20.0)
No	102(51.0)	Uncomfortable	160(80.0)
Level of Education		Feeling comfortable with providing support to the family	
High school	15(7.5)	Comfortable	14(7.0)
Bachelor’s Degree	145(72.5)	Uncomfortable	186(93.0)
Graduate degree	40(20)	Sharing emotions and thoughts after a loss	
Duration of work in the unit		Yes	173(86.5)
< 1 year	29 (14.5)	No	27(13.5)
1–3 years	60(30)	Having enough time after loss	
> 3 years	111(55.5)	Yes	81(40.5)
Willingly working in unit		No	119(59.5)
Yes	155(77.5)	Presence of professional support after loss	
No	45(22.5)	Yes	19(9.5)
		No	181(90.5)

* Only nurses with children were asked to respond

It was observed that 49.0% of the nurses had children and 66.3% of these nurses had never lost their own child, and that 78.0% had not attended any course on the terminal period and the process of grief and that 78.0% had not attended any course on the terminal period and the process of grief. The number of children lost in the units in the last 6 months was 1–10 in 69% of the units. It was seen that 80% of the nurses felt uneasy caring for terminal children and that 93% felt uncomfortable providing the families with emotional support. The data showed that 86.5% of the nurses were comfortable sharing their thoughts and emotions with team members after a loss and that 59.5% said that administrators and colleagues did not give them enough time to get over their loss. It was found that 90.5% of the nurses did not have a professional from whom they could ask for support after a loss.

### Texas Revised Inventory of Grief (TRIG) and Posttraumatic Growth Inventory (PTGI) scores following trauma and related factors

The nurses’ TRIG mean score was 49.425 ± 10.868 (min-max: 17–65) and 61.450 ± 24.934 (min-max: 2-105) on PTGI.

Nurses who had worked in the unit for more than three years (F = 4.617, p = 0.011) and worked willingly (t = 2.120, p = 0.035) were found to have lower TRIG scores. Assessing the nurses by their experiences with loss, it was found that those who had been trained in caring for terminal patients and supporting the patient’s family (t=-2.668, p = 0.001), those who were comfortable with caring for a terminal child (t = 2.624, p = 0.009) and providing support to the child’s family (t = 1.979, p = 0.049), those who lost ten or less children in the unit in the last 6 months (F = 22.507, p = 0.000) and had professional support from the hospital staff (t = 2.797, p = 0.000) had higher TRIG scores, meaning that their level of grief was lower (see Table 2).

**Table 2:** Comparison of TRIG and PTGI scores according to descriptive characteristics (*N = 200*)

Variables	Texas Revised Inventory of Grief				Posttraumatic Growth Inventory			
	Mean ± SD	Test Score	p value	Post hoc	Mean ± SD	Test Score	p value	Post hoc
Age								
21–30 (n = 96)	48.698 ± 11.486	F = 1.259	0.286		58.833 ± 27.092	F = 2.455	0.088	
31–40 (n = 61)	48.934 ± 10.976				60.426 ± 23.512			
Over 40 (n = 43)	51.744 ± 9.056				68.744 ± 20.596			
Gender								
Female (n = 164)	50.195 ± 10.255	t = 2.159	0.069		64.110 ± 23.864	t = 3.299	**0.001**	
Male (n = 36)	45.917 ± 12.896				49.333 ± 26.429			
Civil Status								
Married (n = 108)	49.352 ± 10.512	t=-0.103	0.918		62.861 ± 23.439	t=-0.867	0.392	
Single (n = 92)	49.511 ± 11.328				59.794 ± 26.617			
Level of Education								
High school (n = 15)	49.333 ± 10.527	F = 0.002	0.998		67.800 ± 24.903	F = 3.993	**0.020**	1 > 3, 2 > 3
Undergraduate degree (n = 145)	49.407 ± 10.630				63.428 ± 24.197			
Graduate degree (n = 40)	49.525 ± 12.068				51.900 ± 25.775			
Duration of work in the unit								
< 1 year (n = 29)	51.345 ± 10.801	F = 4.617	**0.011**	2 > 3	60.035 ± 29.770	F = 0.478	0.621	
1–3 years (n = 60)	52.267 ± 8.781				64.083 ± 23.413			
> 3 years (n = 111)	47.387 ± 11.534				60.396 ± 24.489			
Willingly working in unit?								
Yes (n = 155)	48.555 ± 10.840	t = 2.120	**0.035**		64.439 ± 24.216	t = -3.219	**0.002**	
No (n = 45)	52.422 ± 10.537				51.156 ± 24.892			
Has children?								
Yes (n = 98)	50.032 ± 10.173	t=-1.164	0.246		63.929 ± 23.678	t = 1.381	0.169	
No (n = 102)	48.897 ± 11.459				59.069 ± 25.978			
Loss of a child (n = 98)								
Yes (n = 33)	52.849 ± 7.859	t=-1.762	0.081		68.242 ± 23.452	t = 1.289	0.200	
No (n = 65)	49.062 ± 10.911				61.739 ± 23.670			
Education on terminal period and grief?								
Yes (n = 44)	53.227 ± 7.064	t=-2.668	**0.001**		63.273 ± 22.784	t = -0,548	0.584	
No (n = 156)	48.353 ± 11.512				60.936 ± 25.553			
Number of children lost in last 6 months								
1–10 (n = 138)	51.674 ± 9.798	F = 22.507	**0.000**	1 > 3, 2 > 3	64.442 ± 22.509	F = 15.335	**0.000**	1 > 3, 2 > 3
11–20 (n = 36)	49.389 ± 8.926				66.944 ± 27.046			
Over 20 (n = 26)	37.539 ± 11.297				37.962 ± 21.946			
Feeling comfortable looking after a terminal child								
Comfortable (n = 40)	53.400 ± 9.844	t = 2.624	**0.009**		61.575 ± 29.302	t = 0.035	0.972	
Uncomfortable (n = 160)	48.431 ± 10.912				61.419 ± 23.823			
Feeling comfortable while giving the family emotional support								
Comfortable (n = 14)	54.929 ± 11.718	t = 1.979	**0.049**		66.643 ± 31.385	t = 0.807	0.420	
Uncomfortable (n = 186)	49.011 ± 10.721				61.059 ± 24.441			
Sharing emotions and thoughts with team after loss								
Yes (n = 173)	49.867 ± 10.581	t = 1.460	0.066		62.116 ± 23.970	t = 0.955	0.430	
No (n = 27)	46.593 ± 12.398				57.185 ± 30.589			
Being given enough time after the loss								
Yes (n = 81)	51.074 ± 9.468	t = 1.780	0.066		69.667 ± 19.989	t = 3.986	**0.000**	
No (n = 119)	48.303 ± 11.630				55.857 ± 26.452			
Presence of professional support after loss								
Yes (n = 19)	55.947 ± 5.939	t = 2.797	**0.000**		70.579 ± 24.035	t = 1.685	0.094	
No (n = 181)	48.740 ± 11.049				60.492 ± 24.897			

Nurses who had graduate degrees (F = 3.993, p = 0.020), who were female (t = 3.299, p = 0.001) and those who were unwilling to work in the unit (t=-3.219, p = 0.002) had lower PTGI scores. According to their experiences with loss, nurses who lost twenty or more children in the unit in the last six months (F = 15.335, p = 0.000), those who were not given enough time to recuperate by their administrators and teammates (t = 3.986, p = 0.000) had lower PTGI scores (see Table 2).

A significantly positive relationship was found between the TRIG and PTGI overall scores (see Table 3).

**Table 3:** Relationship between TRIG and PTGI scores

	Texas Revised Inventory of Grief Overall Score	
	R	P
Posttraumatic Growth Inventory Overall Score	0.144	***0.041***

### The approaches/applications that nurses thought would be helpful for them after patient loss

A total of 106 participants (53%) responded to the open-ended question. Their responses were reviewed and similar statements were culled together and collected under 9 categories (see Table 4). These categories were: The need for enough time after the loss (n:35); the need for professional support (n:22); the need for social support (n:14); knowing that sufficient care had been given (n:10); the need for training (n:9); not knowing what to do (n:6), leaning towards religion (n:5); avoidance (n:5); continuing with the job (n:2).

**Table 4:** Findings regarding the approaches/applications that nurses thought would be helpful for them after patient loss (*N = 106*)

Opinions and Suggestions	Sample Statements
The need to have enough time to recuperate after the loss (n:35)	Maybe I can be given a half-hour to be by myself so I can question the meaning of life. They could give us some time to get some fresh air so we can blow off steam. I find it hard to care for the other patients soon after a loss; I need to rest for a while. Just to take a break for a short time.
The need for professional support (n:22)	There could be support from a psychologist so we can cope with our emotions. Support from psychologists or people specialized in the field of psychiatry. Psychological support at regular intervals.
The need for social support (n:14)	I would like to get support from the administrators and my teammates. Being in communication with my teammates Spending time with my family
Knowing that sufficient care has been given (n:10)	If I knew that I gave the patient the best care possible, I would feel bad after the loss but I would be happy knowing that the patient was content in the last days of life. Being sure that I gave the child and the family the best care
The need for training (n:9)	Receiving training on how to talk to the family and how to support them. Receiving training to communicate with the family. Being sure that I have done my best. Receiving in-house training in this area
Not knowing what to do (n:6)	I don’t really know. I don’t know.
Leaning towards religion (n:5)	I pray. I remind myself of the reality of death. I read a surah from the Koran.
Avoidance (n:5)	Leaving the patient’s room. Not meeting up with the parents. I wouldn’t want to meet up with the family of a child who died. I would prefer that they saw the body at the morgue.
Continuing with the job (n:2)	Needing to tend to the other children.

Participants who offered more than one suggestion were evaluated under more than one heading

## Discussion

In this study, we looked at pediatric intensive care nurses’ grief process and their posttraumatic growth after a loss, reviewing impacting factors at the same time. The mean score of the PICU nurses on TRIG was 49.43. Since their mean score was higher than 39, it was seen that the intensity of their grief was close to moderate [25, 26]. It has been reported in different studies that pediatric nurses’ experiences with loss are similar and they display a low or moderate level of grief [30, 31]. The PTGI mean score of the pediatric intensive care nurses was found to be 61.45. In studies similar to ours in the literature, it can be seen that nurses mean PTGI score are similar to the level found in our study [32, 33].

In this study, it was found that the higher number of lost children, working longer in the PICU, and wanting to work in the unit increased the grief intensity of nurses. It was reported in one phenomenological study that nurses who experienced greater numbers of patient loss went through a more intense period of grief [34]. Working for a long period in the PICU may lead to experiencing more cases of patient loss. With each loss adding added to the ones experienced before, multiple grief periods and a heavy workload can contribute to a nurse’s experience of intense grieving.

The PICU nurses in the study who had received training on the terminal period and the period of grief, and those who felt comfortable providing the family with emotional support while caring for the child were seen to have lower levels of grief. Similar to our results, Rodriguez et al. [30] showed that receiving education and information on grief produced lower and less intense levels of grief; Plante & Cyr [35] also reported that nurses who were comfortable providing care had lower levels of grief intensity. Providing a dying child and the family emotional support can help nurses to review their own emotions. It was found that one of the approaches the nurses in our study adopted to cope with patient loss was being sure of the quality of the care they provided. Receiving training on the terminal period and the grieving process, as well as supporting both the child and the parents, may help nurses to be more confident of the quality of the care they provide, suffer less stress, and develop appropriate strategies to cope with the loss, leading to less intense periods of grieving. The literature suggests that in order to ensure that healthcare personnel are able to accept patient losses in the pediatric intensive care units, the team should be well-informed and receive regular psychotherapeutic assessments by a psychiatric professional [36]. The present study also showed, similar to the sources in the literature, that receiving support from helpful professionals in the wake of a patient’s loss in their units will contribute to reducing the intensity of grief. Indeed, the nurses used expressions such as “needing a psychologist’s support in coping with our emotions,” “receiving support from specialists in psychology and psychiatry” and “accessing psychological support at regular intervals” in describing their belief that such strategies could help them cope with their situations.

Our study showed greater posttraumatic growth in female nurses. Studies indicate that women demonstrate greater posttraumatic growth compared to men [15, 37–39].

Vishnevsky et al. (2010) explained this difference in their meta-analysis as deriving from the tendency of women to be more deep thinking than men [38]. It is reported that the tendency to reflect on constructive matters such as developing increased awareness about one’s personal strengths or appreciating the importance of social interaction is a mechanism that leads to heightened posttraumatic growth [40]. Another potential mediator in processing traumatic events is coping style [38]. Women reportedly use more emotion-focused coping strategies [41]. Emotion-focused coping involves contemplation, trying to understand an issue, and the cognitive attempt to solve the problem. Emotion-focused coping involves contemplation, trying to understand an issue, and the cognitive attempt to solve the problem thus supporting post-traumatic growth.

Receiving social support has an impact on the coping process as well as on the successful adaptation to traumatic experiences, thus becoming a predictor of posttraumatic growth [42]. In patriarchal societies such as Turkey, men are perceived as strong and primary wage earners. This may act as a barrier to their speaking aloud about their fears and anxieties and asking for professional support. We believe that this may have had an impact on the lesser posttraumatic growth that male nurses displayed.

It has been reported that when there are too many patients lost and no time is allowed for the nurse’s recovery from the trauma, this can be correlated with limited posttraumatic growth among PICU nurses. Nurses who lose numerous patients in intensive care units experience a drop in job satisfaction and consecutive losses may cause nurses to fall into posttraumatic stress disorder [43]. It is these factors that may have caused the nurses to register less posttraumatic growth. The emergence of positive as well as negative factors after a loss may contribute to posttraumatic growth. In our study, the nurses believed that the approach that would help them to best cope with the situation is to be allotted enough time to recover after the trauma. Individuals going through the negative and positive aspects of grief need to have some time to themselves [44]. Our results show that nurses need to be supported in their posttraumatic growth and given time to review their experiences and emotions after suffering patient loss.

There are differing findings in the literature about the relationship between levels of education and posttraumatic growth. Okoli et al. [33] and Pan Cui et al. [45] have reached the conclusion in their studies that those with higher levels of education display greater posttraumatic growth. However, other studies have reported that posttraumatic growth may be associated with lower levels of education [46, 47]. We found in our study as well that nurses with higher education displayed less posttraumatic growth. Bellizzi & Blank (2006) assert that individuals with lesser education may have more areas in which to improve [48]. In other words, individuals with limited education are more liable to turn to different sources for relief from stressful situations. Thus, the issue of whether individuals depend more on their education or on their relationships when dealing with challenges still remains uncertain [49]. Posttraumatic growth includes moral or spiritual growth. [50] Because persons with higher education typically have more of a tendency to question aspects of life, this may be why they display less spiritual growth [33]. This may also be another reason that individuals with graduate education also experience less posttraumatic growth. At the same time, since it is true that the knowledge gained in graduate education does not always tally with real clinical experiences [51], and therefore, this may have had a negative effect on the nurses’ job satisfaction and posttraumatic growth.

Our study showed that nurses willingly working in their hospital unit experienced higher levels of grief and posttraumatic growth. This was not surprising to us. Wanting to work in the unit is a factor that stimulates greater work satisfaction, bringing about the formation of stronger ties with patients and their families, consequently leading to more intensive feelings of grief. A look into the literature reveals studies with results similar to ours in that nurses willingly working in oncology wards exhibit higher levels of posttraumatic growth [32]. No other literature was encountered that explores the relationship between being willing to work in the unit and levels of grief. On the other hand, in a study by Boerner et al. (2015), it was reported that when personnel-patient relations are closer, nurses experience more intense feelings of grief [22].

In this study, we found found that as the grief level of PICU nurses decreased, their post-traumatic growth levels increased. It was reported in a study conducted with certified nurse assistants in a nursing home [9], that nursing assistants with a lesser perception of grief had higher levels of posttraumatic growth. Studies conducted in non-medical populations have reported a negative correlation between grief and posttraumatic growth [46, 52–54]. Gamino et al. (2000) reached the conclusion that anything that increased grief reactions also hindered personal development [52]. Suttle et al. (2022) also found that depression was an important indicator that could be associated with lesser posttraumatic growth [46]. The negative effects of heightened grief on depression and other conditions of mental health may be a barrier to becoming aware of positive feelings such as acknowledging the value of life and living in the present. This may have the effect of delaying or preventing posttraumatic growth.

## Conclusion

According to the results of the study, as nurses’ levels of grief decline, their posttraumatic growth increases. Individuals who experience growth after trauma become more resilient to trauma and therefore more capable of coping with trauma-inducing situations such as grief. This is why it is important to support nurses, such as those in PICU’s, where traumatic experiences are at an intense level, in order to alleviate their grieving process and help them in their posttraumatic growth, to achieve the ease and productivity of the nursing workforce, and to ensure quality patient care.

The posttraumatic growth of PICU nurses following patient loss is affected by many factors that include their individual experiences and their encounters with loss. Our findings show that nurses need training regarding the terminal and grief periods, time to recover before returning to work after a loss and that nurses working in clinics where there are numerous patient deaths, particularly those that have worked in these settings for a long time, need to be regularly supported by mental health professionals. Organizational adjustments to be made by healthcare administrators in the light of these results may contribute to improving the effects of the experiences of nurses after suffering patient loss.

Our findings can be expected to shed light on the grieving process of PICU nurses, their posttraumatic growth levels and impacting factors and we recommend that more qualitative and mixed research is carried out with different groups in an effort to achieve an in-depth understanding of these complex processes.

## Data Availability

The datasets used and/or analyzed during the current study are available from the corresponding author on reasonable request.

## Abbreviations

PICU: Pediatric intensive care unit
TRIG: Texas Revised Inventory of Grief
PTGI: Posttraumatic Growth Inventory
